# Comparative MiRNA Expressional Profiles and Molecular Networks in Human Small Bowel Tissues of Necrotizing Enterocolitis and Spontaneous Intestinal Perforation

**DOI:** 10.1371/journal.pone.0135737

**Published:** 2015-08-14

**Authors:** Pak Cheung Ng, Kathy Yuen Yee Chan, Kam Tong Leung, Yuk Him Tam, Terence Ping Yuen Ma, Hugh Simon Lam, Hon Ming Cheung, Kim Hung Lee, Ka Fai To, Karen Li

**Affiliations:** 1 Department of Paediatrics, Prince of Wales Hospital, The Chinese University of Hong Kong, Shatin, NT, Hong Kong; 2 Department of Surgery, Prince of Wales Hospital, The Chinese University of Hong Kong, Shatin, NT, Hong Kong; 3 Department of Anatomical and Cellular Pathology, Prince of Wales Hospital, The Chinese University of Hong Kong, Shatin, NT, Hong Kong; University of Florida, UNITED STATES

## Abstract

**Background:**

Necrotizing enterocolitis (NEC) and spontaneous intestinal perforation (SIP) are acute intestinal conditions which could result in mortality and severe morbidity in preterm infants. Our objective was to identify dysregulated micro-RNAs (miRNAs) in small bowel tissues of NEC and SIP, and their possible roles in disease pathophysiology.

**Methods:**

We performed differential miRNA arrays on tissues of NEC (n = 4), SIP (n = 4) and surgical-control (Surg-CTL; n = 4), and validated target miRNAs by qPCR (n = 10 each group). The association of target miRNAs with 52 dysregulated mRNAs was investigated by bioinformatics on functional and base-pair sequence algorithms, and correlation in same tissue samples.

**Results:**

We presented the first miRNA profiles of NEC, SIP and Surg-CTL intestinal tissues in preterm infants. Of 28 validated miRNAs, 21 were significantly different between NEC or SIP and Surg-CTL. Limited overlapping in the aberrant expression of miRNAs between NEC and SIP indicated their distinct molecular mechanisms. A proposed network of dysregulated miRNA/mRNA pairs in NEC suggested interaction at bacterial receptor *TLR4* (miR-31, miR-451, miR-203, miR-4793-3p), mediated *via* key transcription factors *NFKB2* (miR-203), AP-1*/FOSL1* (miR-194-3p), *FOXA1* (miR-21-3p, miR-431 and miR-1290) and *HIF1A* (miR-31), and extended downstream to pathways of angiogenesis, arginine metabolism, cell adhesion and chemotaxis, extracellular matrix remodeling, hypoxia/oxidative stress, inflammation and muscle contraction. In contrast, upregulation of miR-451 and miR-223 in SIP suggested modulation of G-protein-mediated muscle contraction.

**Conclusions:**

The robust response of miRNA dysregulation in NEC and SIP, and concerted involvement of specific miRNAs in the molecular networks indicated their crucial roles in mucosa integrity and disease pathophysiology.

## Introduction

Necrotizing enterocolitis (NEC) and spontaneous intestinal perforation (SIP) are the most frequently encountered surgical emergencies, and major causes of morbidity and mortality in preterm infants [1,2]. Despite sharing some common features in clinical presentation, there are major differences between the two conditions, including predisposing factors, radiologic findings, prognosis and natural history of the diseases [3]. The pathophysiology of NEC and SIP has not been fully elucidated, and current knowledge suggests interaction of multiple mechanisms. In a recent study, we reported messenger ribonucleic acid (mRNA) expression profiles of NEC and SIP infants at the tissue level [4]. In NEC tissues, we demonstrated significant dysregulation of 52 target mRNAs categorized under functional pathways of angiogenesis, arginine metabolism, cell adhesion and chemotaxis, extracellular matrix (ECM) remodeling, hypoxia and oxidative stress, inflammation and muscle contraction. These genes could be networked downstream of key receptors: *TLR2*, *TLR4 and TREM1*, and mediated *via* NFκB, AP-1 and *HIF1A* transcription factor pathways, indicating predominant microbial and inflammatory involvement. In contrast, SIP tissues exhibited much milder and less diversified expressional changes with target genes significantly associated with G-protein-mediated muscle contraction and ECM remodeling. Facilitated with the mRNA expression algorithm, we pursue to comprehensively investigate the molecular networks of dysregulation that could be subjected to microribonucleic acid (miRNA) control.

miRNAs are non-coding RNAs of 18–24 nucleotides. They exhibit negative regulation by pairing with complementary sequences in target mRNAs and interfering with their stability and translation [5,6]. miRNAs are synthesized as primary transcripts (pri-miRNAs) and activated *via* two steps of cleavage by specific RNase III enzymes and binding to an argonaute protein [7]. The mature and active miRNA-protein complex will then trigger degradation or block translation of targeted mRNAs. miRNA expressions are known to be tightly regulated in a temporal and tissue-specific manner. To date, over 2,500 mature miRNAs have been reported in humans [8]. Some miRNAs, either acting on self or target cells, are associated with acute and chronic human diseases, including cancer, inflammation, chronic viral infection and acute organ injury [7,9–12]. Aberrant expressions of miRNAs have been reported in ileal and colonic mucosa of inflammatory bowel diseases (IBD) [13–15] and could potentially be used as gut-specific biomarkers for diagnosing and predicting the severity of mucosal injury in these conditions [16–19].

The objectives of the current study were: *(i)* to provide expression profiles of miRNAs in NEC and SIP small bowel tissues, and compared with those of surgical control (Surg-CTL) infants; *(ii)* to validate dysregulated target miRNAs by quantitative polymerase chain reaction (qPCR) assay; and *(iii)* to identify potential association of miRNAs with dysregulated mRNAs using bioinformatics algorithms and correlation analysis of target miRNA/mRNA pairs in same tissue specimens, so as to reveal possible mechanisms of miRNAs in the disease pathophysiology. To our knowledge, the miRNA profile or its regulatory mechanism has not been reported in NEC or SIP at the tissue or blood level in preterm infants. As miRNAs are emerging as a new class of blood-based biomarkers and therapeutic targets, [13,20] our findings could provide crucial information concerning their functional roles in NEC and SIP, which could further shed light on their clinical applications.

## Materials and Methods

### Patients and Sample Collection

In this case control study, small bowel specimens from 3 categories of infants were collected: *(i)* NEC, *(ii)* SIP and *(iii)* surgical control (Surg-CTL) cases. Infants with stage III NEC (n = 10) and SIP (n = 10) who underwent surgery were recruited at the Prince of Wales Hospital, The Chinese University of Hong Kong [4]. In brief, intestinal specimens were collected at the time of surgery and all NEC and SIP cases were proven by histology. For each NEC specimen, a sample of inflamed but non-necrotic tissue was dissected from an area 3–5mm from the margin of the resected bowel. SIP is defined as an isolated perforation of the small bowel, common in the ileum and with no obvious macroscopic inflammation in the adjacent site or the rest of the bowel. The perforation was either resected with primary anastomosis (n = 1) or with construction of a temporary ileostomy (n = 9). Similar to NEC cases, intestinal tissues were collected from the margin of the perforation site of SIP infants. Surg-CTL tissues were obtained from infants who underwent intestinal surgery (n = 10) because of: *(i)* congenital small bowel atresia (n = 7), *(ii)* intestinal obstruction [ileal stenosis (n = 1) and meconium ileus (n = 1)], and *(iii)* elective closure of ileostomy with tissue specimens collected at the functional end of the small bowel (n = 1). All Surg-CTL infants had primarily non-inflammatory intestinal conditions. The demographic information of the 3 groups of infants is described in Table 1.

**Table 1:** Clinical Characteristics of NEC, SIP and Surg-CTL Infants.

Infants	NEC	SIP	Surg-CTL	NEC vs Surg-CTL, *P*	SIP vs Surg-CTL, *P*	NEC vs SIP, *P*
No. of infants	10	10	10	-	-	-
Sex, female	5 (50%)	2 (20%)	5 (50%)	1.000	0.350	0.350
Gestational age, wk	28.9 (26.3–34.7)	26.5 (24.1–29.0)	34.1 (32.3–37.9)	0.082	**0.007**	0.104
Birthweight, g	1080 (821–2090)	884 (695–1403)	2113 (1813–2773)	0.070	**0.013**	0.364
Apgar scores:						
1 min	7 (4–8)	5 (4–6)	8 (7–9)	0.129	**0.002**	0.194
5 min	8 (7–10)	7 (6–8)	9 (9–9)	0.086	**0.002**	0.535
Infants received enteral feeding before surgery, n	10 (100%)	4 (40%)	5 (50%)	0.033#	1.000	**0.011**
Age commenced on feeding, d	3 (1–4)	11 (9–22)	10 (2–16)	0.267	0.177	**0.003**
Age of full enteral feeding, d	31 (18–89)	35 (30–82)	35 (13–77)	0.825	0.401	0.507
Postnatal age at the onset of illness, d	29 (15–45)	9 (2–12)	3 (1–78)	0.129	0.340	**0.006**
Drug administrated before surgery:						
Indomethacin or ibuprofen	4 (40%)	6 (60%)	0 (0%)	0.087	**0.011**	0.656
Systemic corticosteroids	1 (10%)	1 (10%)	0 (0%)	1.000	1.000	1.000
Antibiotics	10 (100%)	10 (100%)	6 (60%)	0.087	0.087	1.000
Duration of hospitalization, d	205 (134–251)	127 (88–157)	91 (30–182)	**0.014**	0.450	0.028#
Duration of disease onset to surgery, hr	48 (29–64)	7 (5–10)	23 (13–35)	0.061	**0.002**	**<0.001**
Length of bowel resection, cm	35 (23.0–40.5)	4.5 (2.4–10.1)	4.8 (3.4–18.4)	**0.002**	0.404	**<0.001**
No. died	3	1	1	0.582	1.000	0.582

Results are expressed as number (%) or median (interquartile range). Standard antibiotic regimens in our neonatal unit for treatment of NEC and SIP, included: (i) vancomycin, aminoglycoside or 3rd generation cephalosporin and metronidazole, or (ii) vancomycin and meropenem, depending on the severity of intra-abdominal pathology and microbial culture/sensitivity. Bold values indicate statistical significance (*P*<0.016) after the Bonferroni correction.

^***#***^ Comparison not significant after the Bonferroni correction.

### Ethical Consideration

Written parental consent was obtained for all cases and this study has been approved by the Joint Chinese University of Hong Kong and New Territories East Cluster Clinical Research Ethics Committee.

### Tissue Preparation, miRNA Analysis and Validation of Expression by qPCR

Collected specimens were immediately rinsed with cold diethyl pyrocarbonate-treated phosphate-buffered saline, snap-frozen in liquid nitrogen and stored at -80°C. All specimens were processed and analyzed independently. RNA was extracted using TRIZOL reagent (Life Technologies, Gaithersburg, MD) and miRNeasy kit (QIAGEN, GmbH, Hilden, Germany). The total RNA concentration was measured using a Nanodrop ND-1000 Spectrophotometer (NanoDrop Technologies Inc., Wilmington, DE).

For microarray analysis, 400ng of RNA from each specimen was labelled with the FlashTag Biotin RNA Labelling Kit and hybridized to GeneChip miRNA array covering 1,733 human mature miRNAs (Affymetrix, Santa Clara, CA). Genechips were then washed and stained using the FS450 Fluidic Station (Affymetrix). The fluorescent signal intensity of stained chips was captured by the GeneChip Scanner 3000 7G System (Affymetrix). Data were analyzed using the Partek Genomics Suite v6.5 software (Partek, St. Louis, MO). Probeset signals were normalized with the Robust Multi-array Average method and log_2_-transformation. miRNA profiles for NEC (n = 4), SIP (n = 4) and Surg-CTL (n = 4) tissues were generated and compared among the three groups *i*.*e*., NEC *vs*. Surg-CTL, SIP *vs*. Surg-CTL, and NEC *vs*. SIP. Experimental details have been submitted to Gene Expression Omnibus (GEO) under the series accession number GSE68054. Selection of miRNAs for subsequent validation by qPCR was achieved using the following criteria: *(i)* ≥2-fold with significant difference (*P*<0.05) between NEC or SIP and Surg-CTL, *(ii)* potential target genes being within the list of 52 dysregulated genes reported in NEC or SIP tissues,[4] and/or *(iii)* reported association with the gastrointestinal (GI) tract, IBD or inflammatory responses. The potential miRNA/mRNA pairs were identified by multiple target prediction algorithms based on sequence matching and functional reports.

qPCR analysis was performed on NEC (n = 10), SIP (n = 10) and Surg-CTL (n = 10) tissues, inclusive of those collected at the earlier time frame or microarray analysis (n = 4 for each group). cDNA was prepared from 10ng of total RNA from each specimen using the TaqMan MicroRNA Reverse Transcription Kit and TaqMan MicroRNA Assay containing pre-designed primers for selected miRNAs (S1 Table). qPCR reactions were performed in duplicate with cDNA (2μL), 2xTaqMan Universal PCR Master Mix (10μL), and specific primers and probes (final volume 20μL). Amplification was performed for 40 cycles with denaturation at 95°C for 15sec, and annealing extension at 60°C for 1min. The emission intensity was detected by the ABI 7300 Real-Time PCR System (Life Technologies). U6 snRNA was used as the reference gene for normalization, as indicated by the optimization and selection data (S1 Fig).

### Target mRNA Prediction and Correlation of Dysregulated miRNA and mRNA Pairs

Putative mRNA targets of dysregulated miRNAs were searched amongst the 52 significantly changed mRNAs identified in our previous study of NEC and SIP tissues using 5 databases: target sequence prediction DIANA-microT web server v5.0, [21] Tarbase 6.0, [22] MicroCosm (http://www.ebi.ac.uk/enright-srv/microcosm/htdocs/targets/v5/), TargetScan 6.2 [23] and network analysis software Metacore Analysis Suite (GeneGo-Thomson Reuters, St. Joseph, MI). The correlation coefficient on levels (qPCR) of specific miRNAs and their predicted target mRNAs in same tissue samples of NEC or SIP were assessed (n = 10).

### Construction of Interactive Networks on Dysregulated miRNAs and mRNAs

Selection of miRNA and sequence-matched mRNA pairs for network analysis was based on two criteria: *(i)* miRNA and mRNA [4] were dysregulated at opposite directions (*i*.*e*., significantly increased miRNA and decreased mRNA, or *vice versa*) with reference to their respective Surg-CTLs, and/or *(ii)* a significant inverse correlation between levels of miRNA and target mRNA in same tissue specimens. The association of these target miRNAs in the functional pathways of dysregulated mRNAs was illustrated in a network diagram generated by the MetaCore software.

### Statistical Analysis

The demographic and clinical characteristics of NEC, SIP and Surg-CTL infants were compared using the Fisher’s exact test and Mann-Whitney *U* test, where appropriate. Expression levels of target miRNAs between NEC *vs*. Surg-CTL, SIP *vs*. Surg-CTL or NEC *vs*. SIP, were compared using the Mann-Whitney *U* test. The Bonferroni correction was applied for multiple group comparisons and a *P* value of <0.0167 was considered as statistically significant. The correlation between the levels of specific miRNAs with corresponding dysregulated mRNAs in same specimens was computed using the Spearman’s correlation test. Results are expressed as median and interquartile range, or as mean fold change. All analyses were performed using SPSS (Version 21, Chicago, IL).

## Results

### Clinical Characteristics of Study Infants

The clinical characteristics of NEC, SIP and Surg-CTL infants are summarized in Table 1. As expected, infants with NEC were significantly different from SIP with regard to their postnatal age at the onset of illness, parameters related to commencement of enteral feeding, duration of disease onset to surgery and length of bowel resection. Also, NEC infants had longer duration of hospitalization and length of bowel resection, compared with Surg-CTL infants. Due to different nature of the conditions, SIP infants exhibited different clinical demographics, use of non-steroidal anti-inflammatory drugs (*i*.*e*., indomethacin or ibuprofen), and duration of disease onset to surgery, compared with Surg-CTL infants. One Surg-CTL patient died from VATER Syndrome, which was independent of gut abnormality.

### miRNA Microarray Profiles in Small Bowel Tissues of NEC, SIP and Surg-CTL

**Fig 1:**
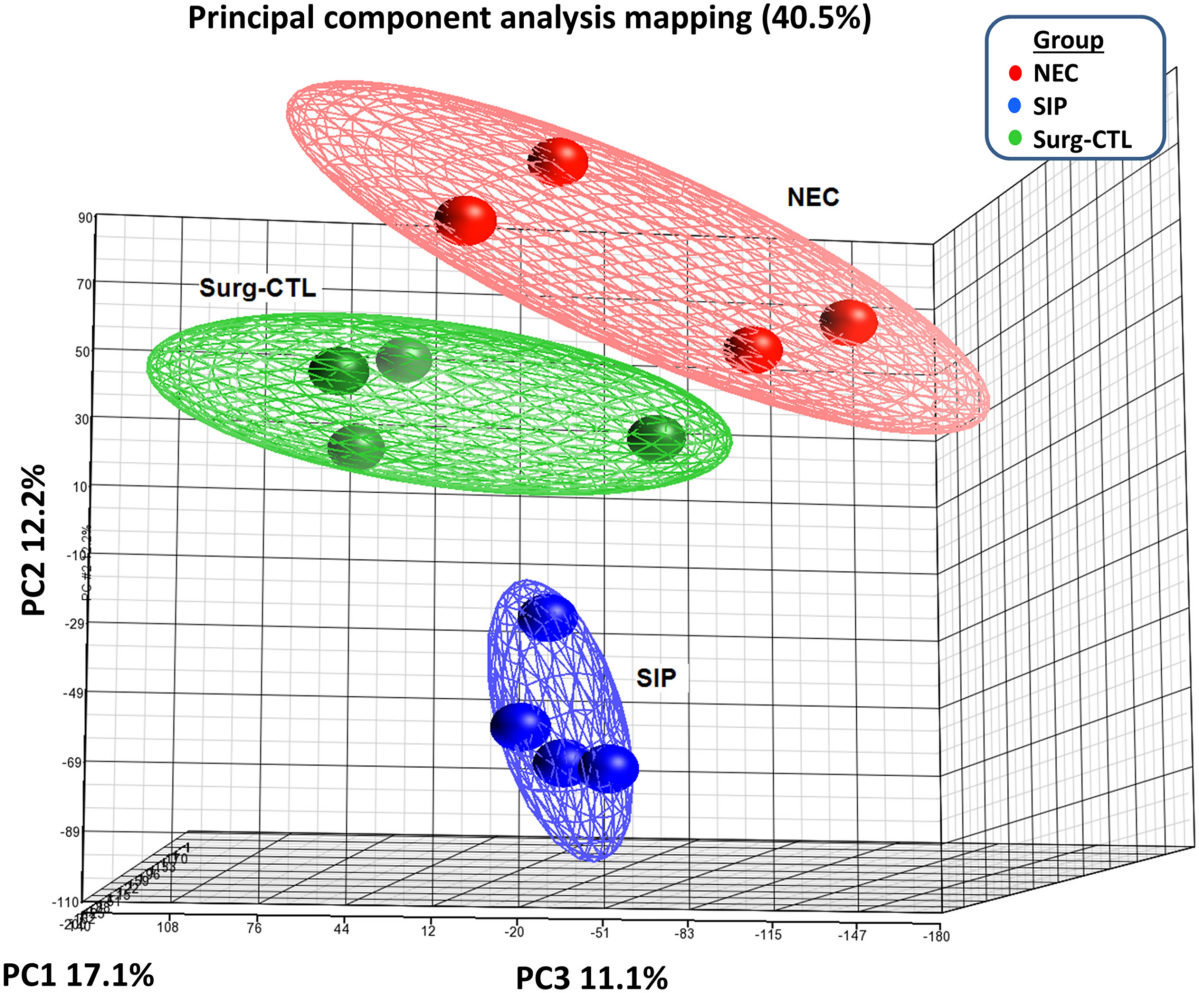
Principal component analysis of miRNA expression profiles in NEC, SIP and Surg-CTL small bowel tissues. miRNA expression profiles of NEC (n = 4), SIP (n = 4) and Surg-CTL (n = 4) tissues were analyzed by principal component analysis using the Partek Genomics Suite. The ellipsoids represent 95% confidence intervals of NEC (red), SIP (blue) and Surg-CTL (green) clusters. Each dot represents an experiment dataset. The axes correspond to principal component 1 (PC1; x-axis), PC2 (y-axis) and PC3 (z-axis).

Analysis of NEC, SIP and Surg-CTL miRNA profiles revealed tight clustering of gene expression datasets within each groups, with distinct separation amongst NEC, SIP and Surg-CTL (Fig 1). NEC demonstrated significant up- and down-regulation of 80 miRNAs (2.01–33.22 fold) and 27 miRNAs (0.003–0.047 fold) compared with Surg-CTL, respectively. In addition, 73 (2.00–7.57 fold) and 18 (0.12–0.50 fold) miRNAs were significantly up- and down-regulated in SIP *vs*. Surg-CTL. The comparison of microarray data on selected miRNAs from NEC, SIP and Surg-CTL tissues is described in S2 Table.

### Validation of Differentially Expressed miRNAs in NEC and SIP by qPCR

Of 28 selected differentially expressed miRNAs, 21 were validated by qPCR as statistically different between NEC (n = 10) or SIP (n = 10) and Surg-CTL (n = 10), or between NEC and SIP tissues (*P*<0.0167; Table 2). Validated miRNAs could be classified into 3 categories:

1. *NEC-associated miRNAs*: 10 miRNAs *i*.*e*., miR-223, miR-451, miR-1290, miR-4725-3p, miR-431, miR-4793-3p, miR-21-3p, miR-132, miR-146b-3p and miR-410 were significantly increased in NEC compared with Surg-CTL tissues (3.36–25.16 fold), whereas 9 miRNAs: miR-375, miR-203, miR-200b-5p, miR-194-3p, miR-200a, miR-215, miR-31, miR-192-3p and miR-141 were significantly decreased in NEC tissues (0.07–0.31 fold). Levels of 15 miRNAs: miR-223, miR-1290, miR-4725-3p, miR-4793-3p, miR-410, miR-187, miR-375, miR-203, miR-200b-5p, miR-194-3p, miR-200a, miR-215, miR-31, miR-192-3p and miR-141 were significantly different between NEC and SIP tissues (0.12–59.05 fold; Table 2).
2. *SIP-associated miRNAs*: 5 miRNAs *i*.*e*., miR-223, miR-451, miR-431, miR-132 and miR-429 were significantly increased in SIP compared with Surg-CTL tissues (1.80–8.02 fold). Except miR-429, these miRNAs were also increased in NEC (4.88–25.16 fold; Table 2).
3. *miRNAs not different among tissue groups*: 7 miRNAs *i*.*e*., miR-1231, miR-1, miR-602, miR-4440, miR-133b, miR-23b-5p and miR-490 were expressed in tissues of NEC, SIP and Surg-CTL. Yet, no significant differential levels were demonstrated among tissue groups (Table 2).

**Table 2:** Differentially Expressed miRNAs in Small Bowel Tissues from Infants with NEC (n = 10), SIP (n = 10) and Surg-CTL (n = 10) by qPCR Assay.

	NEC vs Surg-CTL		SIP vs Surg-CTL		NEC vs SIP	
	Fold	*P*	Fold	*P*	Fold	*P*
miR-223	25.16	**0.000**	3.23	**0.002**	7.78	**0.001**
miR-451	22.77	**0.003**	8.02	**0.013**	2.84	0.650
miR-1290	12.88	**0.001**	0.22	0.326	59.05	**0.000**
miR-4725-3p	8.85	**0.005**	0.80	0.623	11.08	**0.003**
miR-431	7.05	**0.002**	4.15	**0.007**	1.70	0.450
miR-4793-3p	6.00	**0.007**	0.14	0.406	42.35	**0.002**
miR-21-3p	5.17	**0.000**	1.95	0.096	2.65	**0.017#**
miR-132	4.88	**0.001**	3.74	**0.010**	1.31	0.326
miR-146b-3p	4.12	**0.001**	1.84	0.059	2.24	**0.049#**
miR-410	3.36	**0.003**	1.20	0.545	2.79	**0.013**
miR-429	1.56	**0.174**	1.80	**0.013**	0.86	0.450
miR-187	1.05	**0.545**	0.39	**0.028#**	2.71	**0.007**
miR-375	0.31	**0.002**	1.06	0.762	0.30	**0.003**
miR-203	0.31	**0.004**	1.30	0.940	0.24	**0.005**
miR-200b-5p	0.29	**0.003**	1.00	0.791	0.29	**0.003**
miR-194-3p	0.28	**0.002**	1.12	0.597	0.25	**0.004**
miR-200a	0.20	**0.002**	0.78	0.290	0.25	**0.003**
miR-215	0.13	**0.001**	0.72	0.762	0.18	**0.002**
miR-31	0.12	**0.001**	0.52	**0.041#**	0.23	**0.002**
miR-192-3p	0.11	**0.001**	0.81	0.290	0.14	**0.001**
miR-141	0.07	**0.001**	0.64	0.112	0.12	**0.001**
miR-1231	3.67	**0.049#**	0.82	0.880	4.50	**0.028#**
miR-1	2.08	0.406	0.64	0.290	3.25	0.070
miR-602	1.94	0.364	2.08	0.326	0.93	0.496
miR-4440	1.90	0.070	1.37	0.227	1.39	0.706
miR-133b	1.88	0.597	0.64	0.364	2.92	0.070
miR-23b-5p	1.65	0.112	0.64	0.257	2.58	**0.023#**
miR-490	1.21	0.345	1.34	0.112	0.90	0.059

Bold values indicate statistical significance *(P* < 0.0167).

^***#***^ Comparison not significant after Bonferroni correction.

A literature research on selected miRNAs, their potential target genes and relevant functions is summarized in S3 Table.

### Predicted Association of Dysregulated miRNAs and Target mRNAs

Bioinformatics analysis was performed for mapping putative mRNA targets by base-pair sequence and functional association between significantly altered miRNAs and 52 dysregulated mRNAs in NEC and SIP tissues reported in our previous study [4]. The matched miRNA/mRNA pairs are listed in S4 Table. Our results showed that 35 miRNA/mRNA pairs (49%) in NEC were dysregulated in opposite directions with respect to Surg-CTL (*i*.*e*., increased miRNA and decreased mRNA or vice versa), whereas 37 pairs (51%) were changed in the same direction (*i*.*e*., both miRNA and mRNA were increased or decreased). In SIP, 3 pairs (75%) of miRNA/mRNA were dysregulated in an opposite direction, whereas one pair (25%) was changed in the same direction.

**Fig 2:**
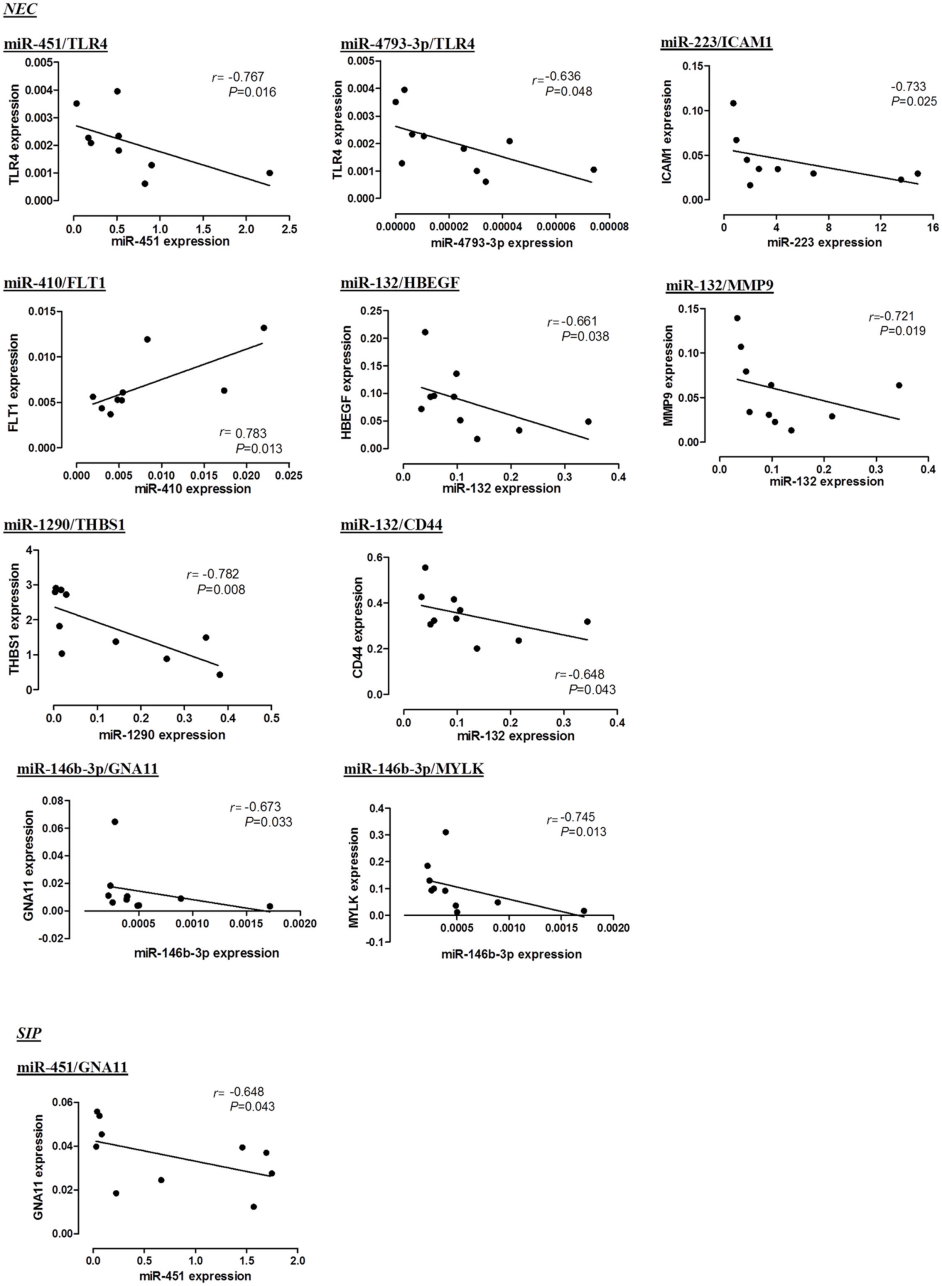
Correlation of dysregulated miRNAs with potential target mRNAs in NEC and SIP tissues. The linear regression coefficient (*r*) of qPCR levels of miRNAs and respective target mRNAs in NEC (n = 10) or SIP (n = 10) tissues were analyzed using the Spearman’s correlation test.

Based on qPCR levels of miRNAs and respective target mRNAs in same disease specimens, we investigated their correlation in NEC or SIP tissues. Our results showed that levels of miRNA/mRNA pairs: miR-451/*TLR4*, miR-4793-3p/*TLR4*, miR-132/*HBEGF*, miR-1290/*THBS1*, miR-132/*CD44*, miR-223/*ICAM1*, miR-132/*MMP9*, miR-146-3p/*GNA11* and miR-146-3p/*MYLK* were significantly correlated in an inverse manner in NEC tissues, whereas miR-410/*FLT-1* was directly correlated (Fig 2). In SIP tissues, miR-451/*GNA11* was inversely correlated.

### Regulatory Molecular Networks in NEC and SIP

**Fig 3:**
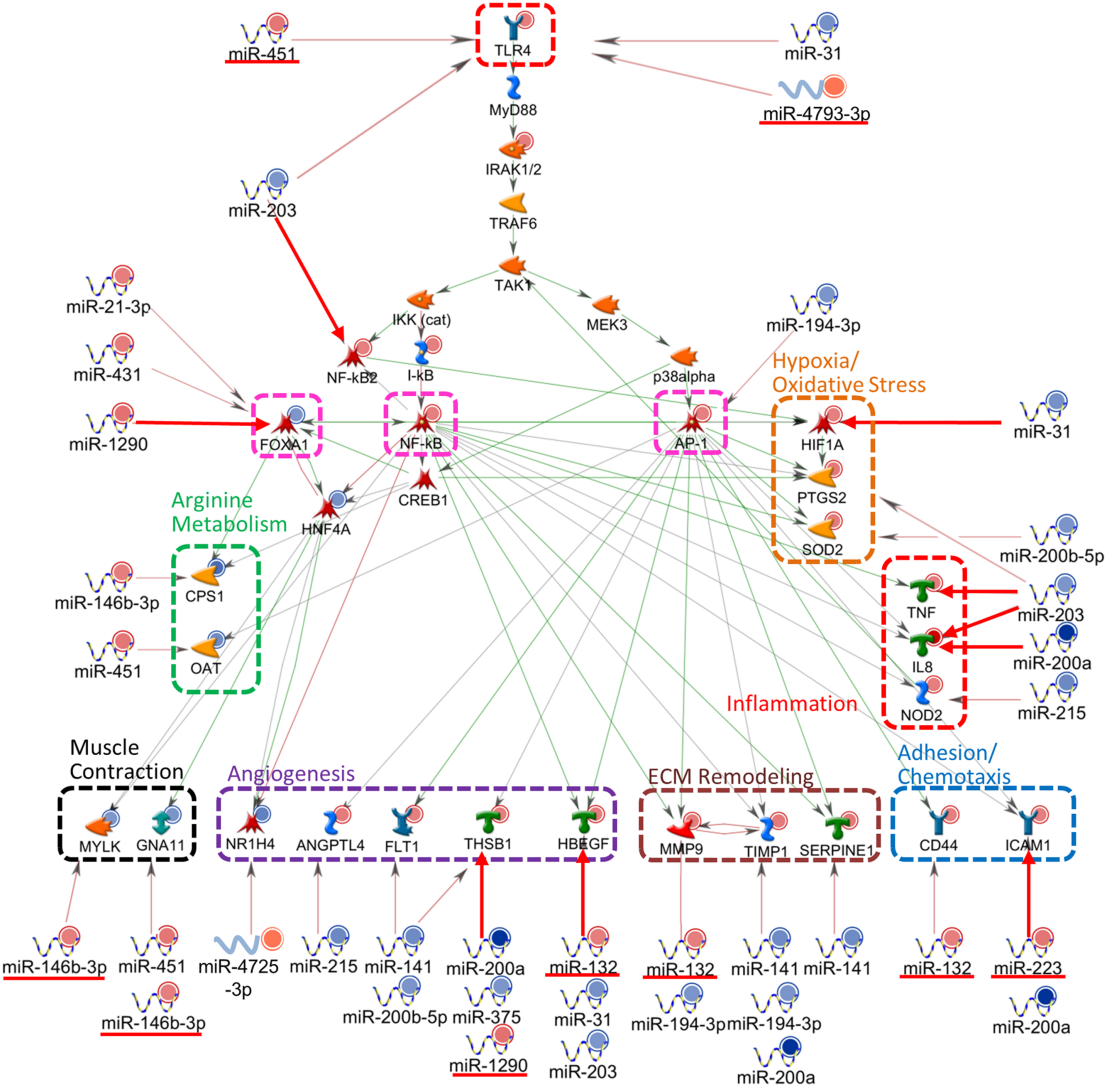
Pathway traced network of miRNA/mRNA pairs in NEC. A functional network on the association of selected miRNAs and potential target mRNAs was generated by the MetaCore software and sequence-based prediction. These miRNA/mRNA pairs were regulated at opposite directions and/or exhibited significant correlation in same samples. qPCR-validated expression changes of miRNAs or mRNAs are shown as red and blue color circles, representing up- and down-regulation. Green, red and grey arrows between target genes represent positive, negative and unspecified interactions, respectively. Big arrows between miRNA/mRNA pairs indicate experimentally proven relationships. Transcription factors and functional categories, including angiogenesis, arginine metabolism, cell adhesion, chemotaxis and inflammation, ECM remodeling, hypoxia/oxidative stress, and muscle contraction are highlighted in different colors. miRNAs which exhibited significant inverse correlation with specific mRNAs are underlined.

The proposed network in NEC indicated miRNA involvement directed at multiple levels, including upstream receptor: *TLR4* (miR-31, miR-203, miR-451, miR-4793-3p), mediated *via* transcription factors: *NFKB2* (miR-203), AP-1*/FOSL1* (miR-194-3p), *FOXA1* (miR-21-3p, miR-431, miR-1290) and *HIF1A* (miR-31), as well as downstream functional regulators of: angiogenesis (miR-31, miR-132, miR-141, miR-200a, miR-200b-5p, miR-203, miR-215, miR-375, miR-1290, miR-4725-3p), arginine metabolism (miR-146b-3p, miR-451), cell adhesion and chemotaxis (miR-132, miR-200a, miR-223), ECM remodeling (miR-132, miR-141, miR-194-3p, miR-200a,), hypoxia and oxidative stress (miR-31, miR-200b-5p, miR-203), inflammation (miR-200a, miR-203, miR-215) and muscle contraction (miR-146-3p, miR-451) (Fig 3).

**Fig 4:**
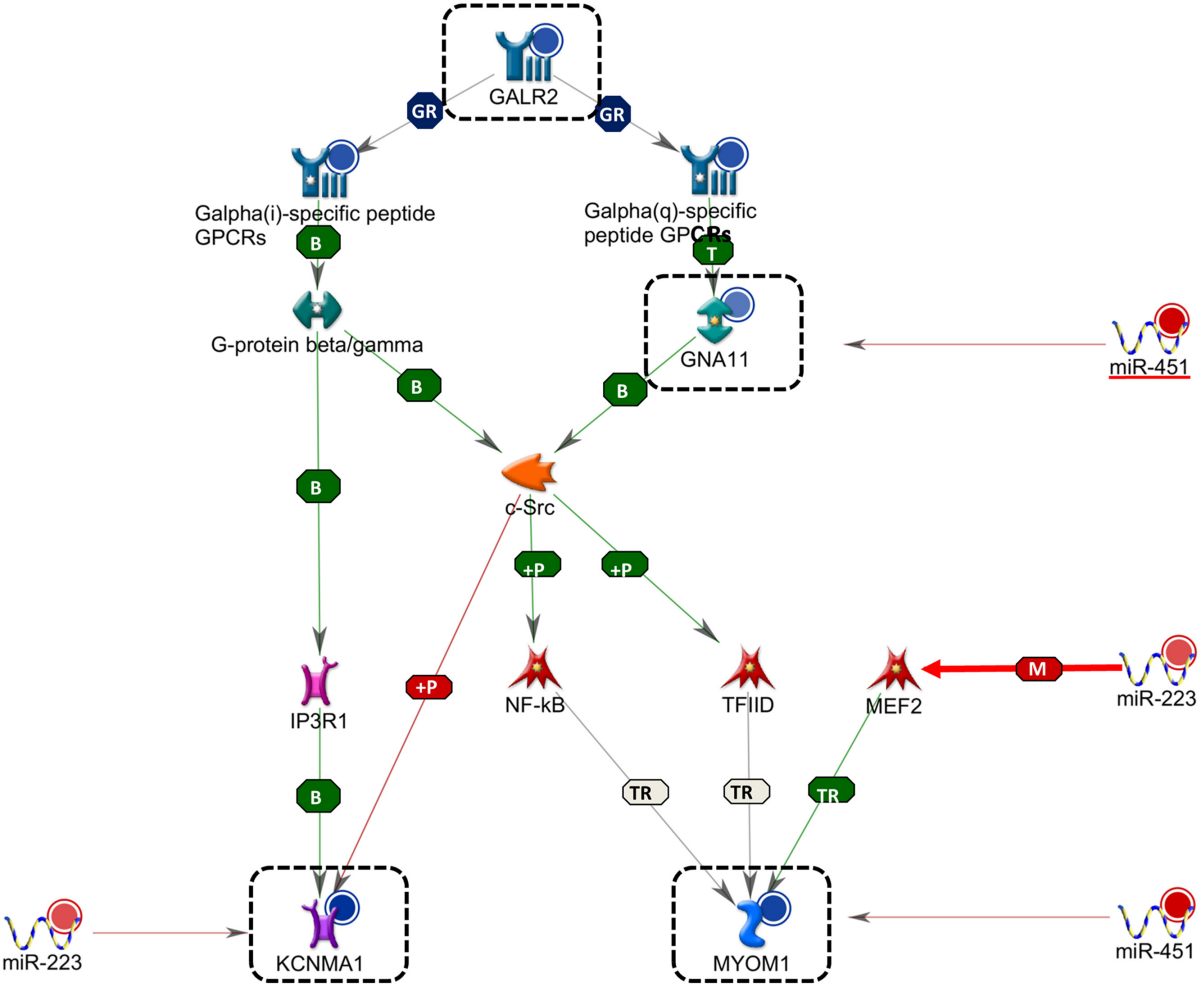
Interacting network of miRNA/mRNA pairs in SIP. A molecular network of miRNA/mRNA regulation associated with G-protein-mediated muscle contraction is illustrated in the diagram. Interactions between genes are indicated as binding (B), group relationship (GR), miRNA binding (M), phosphorylation (+P), transformation (T) and transcriptional regulation (TR). Expression changes are shown as red and blue color circles, representing up- and down-regulation. Green, red and grey arrows between target genes represent positive, negative and unspecified interactions, respectively. Big arrows between miRNA/mRNA pairs indicate experimentally proven relationships.

In SIP tissues, miR-223 and miR-451 were up-regulated and their potential target genes *KCNMA1*, *GNA11*, *MYOM1* were down-regulated compared with Surg-CTL tissues (Table 3). Expressions of miR-451 and *GNA11* were also significantly and inversely correlated. A molecular network of miRNA/mRNA regulation associated with G-protein-mediated muscle contraction is shown in Fig 4.

**Table 3:** miRNA and mRNA Targets in Regulatory Networks of NEC and SIP.

Predicted mRNA targets	miRNAs
**NEC**	
Receptors	
*TLR4* (↑)	miR-451 (↑)*, miR-4793-3p (↑)*, miR-31 (↓), miR-203 (↓)
Transcription Factors and Network Intermediates	
*FOSL1* (↑)	miR-194-3p (↓)
*FOXA1* (↓)	miR-21-3p (↑), miR-431(↑), miR-1290 (↑)
*NFKB2* (↑)	miR-203 (↓)
Angiogenesis	
*ANGPTL4* (↑)	miR-215 (↓)
*FLT1* (↑)	miR-141 (↓), miR-200b-5p (↓)
*HBEGF* (↑)	miR-132 (↑)*, miR-31 (↓), miR-203 (↓)
*NR1H4* (↓)	miR-4725-3p (↑)
*THBS1* (↑)	miR-1290 (↑)*, miR-141 (↓), miR-200a (↓), miR-375 (↓)
Arginine Metabolism	
*CPS1* (↓)	miR-146b-3p (↑)
*OAT* (↓)	miR-451 (↑)
Cell Adhesion and Chemotaxis	
*CD44* (↑)	miR-132 (↑)*
*ICAM1* (↑)	miR-223 (↑), miR-200a (↓)
ECM Remodeling	
*MMP9* (↑)	miR-132 (↑)*, miR-194-3p (↓)
*SERPINE1* (↑)	miR-141 (↓)
*TIMP1* (↑)	miR-194-3p (↓), miR-200a (↓), miR-141 (↓)
Hypoxia and Oxidative Stress	
*HIF1A* (↑)	miR-31 (↓)
*PTGS2* (↑)	miR-203 (↓)
*SOD2* (↑)	miR-200b-5p (↓)
Inflammation	
*IL8* (↑)	miR-200a (↓), miR-203 (↓)
*NOD2* (↑)	miR-215 (↓)
*TNF* (↑)	miR-203 (↓)
Muscle Contraction	
*GNA11* (↓)	miR-146b-3p (↑)*, miR-451 (↑),
*MYLK* (↓)	miR-146b-3p (↑)*
**SIP**	
Muscle Contraction	
*KCNMA1* (↓)	miR-223 (↑)
*GNA11* (↓)	miR-451 (↑)*
*MYOM1* (↓)	miR-451 (↑)

qPCR-confirmed significant over-expression (↑) and under-expression (↓) in NEC or SIP tissues compared with Surg-CTL tissues.

*Significant inverse correlation (*P*<0.05) of miRNA/mRNA pairs in same NEC or SIP specimens.

## Discussion

To date, very limited information has been reported on the role of the miRNA mechanism in preterm infants, of whom the innate immunologic response is known to be immature and compromised. As far as we are aware, this is the first study reporting profiles of miRNA expression in NEC and SIP infants at the tissue level, and their possible roles in affecting downstream circuits of gene expression and regulatory pathways. The strength of this study is that we have access to our own established miRNA and mRNA microarray databases, and qPCR-validated levels of miRNAs and putative target mRNAs from the same NEC, SIP and Surg-CTL specimens, in addition to web-based bioinformatics algorithms. The collective information allowed us to focus the network criteria on miRNA/mRNA pairs with quantitative evidence and functional relevance. In NEC tissues, we identified target miRNAs which could potentially act on the key receptor *TLR4*, transcription factors NFκB/*NFKB2*, AP-1*/FOXA1*, *HIF1A* and downstream effector genes in functional networks of angiogenesis, arginine metabolism, cell adhesion/chemotaxis, ECM remodeling, hypoxia/oxidative stress inflammation and muscle contraction, whereas dysregulated miRNAs in SIP tissues were mainly directed towards mRNAs associated with G-protein mediated muscle contraction. The limited change of miRNAs in SIP tissues and minimal overlapping between aberrant miRNA expressions in NEC and SIP further supported that the two conditions are distinct diseases that exhibited different molecular mechanisms and pathophysiology [4].

Our findings suggested that specific miRNAs could exert influence on NEC tissues in an organized manner, directed at bacterial receptor *TLR4* and extended to involve multiple downstream pathways of key functional genes. These results indicated the important roles of miRNAs in modulating the inflammatory response, which is likely mediated by etiologic factors such as bacterial invasion and hypoxia/oxidative stress. When miRNAs such as miR-31 and miR-203 were down-regulated in NEC tissues, they could potentiate the upsurge of proinflammatory signals mediated by *TLR4*, key transcription factor *NFκB*, hypoxia/oxidative regulators *HIF1A* and *PTGS2*, inflammatory cytokines and chemokines *TNF* and *IL8*, as well as angiogenic factor *HBEGF*, resulting in extensive mucosal injury (Fig 3). Interestingly, we identified miRNAs that could exhibit multiple interacting points at the upstream receptor and transcription factors as well as downstream effector genes *e*.*g*., miR-31 (*TLR4*, *HIF1A*, *HBEGF*), miR-194-3p (AP-1*/FOSL1*, *MMP9*, *TIMP1*), miR-203 (*TLR4*, *NFKB2*, *HBEGF*, *IL8*, *TNF*, *PTGS2*), miR-1290 (*FOXA1*, *THBS1*) and miR-200b-5p (*SOD2*, *FLT1*). Other miRNAs could be more specific, directing at single genes *e*.*g*., miR-4793-3p (*TLR4*), miR-431 (*FOXA1*) and miR-375 (*THBS1*). We speculate that these observations reflected a concerted miRNA response in the neonatal gut that could encompass efficiency of a single miRNA acting at multiple levels of the regulatory cascade, as well as fine-tuning the mechanism by directing a specific miRNA at one target mRNA. In addition, we observed that some miRNA/mRNA pairs were dysregulated at same directions *i*.*e*., both miRNA and mRNA were significantly increased or decreased with respect to Surg-CTL tissues. Yet, their levels were inversely correlated within same NEC samples such as miR-451/*TLR4* (receptor), miR-4793-3p/*TLR4* (receptor), miR-132/*HBEGF* (angiogenesis), miR-1290/*THBS1* (angiogenesis), miR-132/*CD44* (adhesion/chemotaxis) and miR-132/*MMP9* (ECM remodeling). This apparently paradoxical phenomenon suggested that these miRNAs might have exerted inhibitory effects on their target mRNAs at various points of the inflammatory cascade, but their magnitudes were insufficient to reverse or nullify the overall mRNA response in the pathologic process.

Some dysregulated miRNAs such as miR-1290, miR-146b-3p, miR-31, miR-375 and miR-200a have established GI functions (S3 Table). Alternatively, miRNAs can be secreted from infiltrating leukocytes into the microenvironment of matrix, neighboring gut endothelium and muscle cells, as well as into the circulation. Thus, they could act as efficient mediators to provide organized responses across cell barriers. This mechanism would likely be prominent in NEC tissues where inflammatory neutrophils and macrophages are in abundance. For example, miR-203 could target *myd88* in macrophages against LPS or bacterial infection [24]. miR-451 targeting *CPNE3* and *RAB5A*, [25] and miR-141 targeting *CXCL12B* [26] have known functions on leukocyte chemotaxis and migration [26]. In the current study, miR-451 was inversely correlated with *TLR4*, and could also regulate arginine hemostasis (*OAT*) and G-protein mediated muscle contraction (*GNA11*). miR-141 could potentially activate angiogenesis (*FLT1*, *THBS1*) and ECM remodeling (*TIMP1*, *SERPINE1*) by reducing the inhibitory effect on target mRNAs. Our results also revealed a group of dysregulated miRNAs, including miR-1290, miR-431, and miR-200a that might interfere with cell proliferation and differentiation (S3 Table), indicating responses that could lead to tissue injury and attempted repair. Overall, specific miRNAs could dictate important functional pathways in the pathophysiology of NEC by interfering with the inflammatory cascade and gut functions, in response to bacterial invasion and hypoxia/oxidative stress.

In SIP tissues, the regulation of miRNA/mRNA pairs, including miR-223/*KCNMA1*, miR-451/*GNA11* and miR-451/*MYOM1* indicated involvement of these miRNAs in dysregulating the muscle contraction pathway, a major pathologic mechanism associated with SIP [4]. Our network analysis demonstrated that the increased expression of miR-223 and miR-451 could lead to upstream inhibition of G-protein *GNA11*, and in turn down-regulation of the ion channel regulator *KCNMA1* and muscle contractile gene *MYOM1 via* a tyrosine-protein kinase *SRC* and NF-κB signaling (Fig 3). miR-223 and miR-451 have known functions on proliferation and differentiation of vascular and skeleton muscles, [27,28] but their novel involvement in neonatal gut muscle physiology and SIP pathophysiology has not been reported previously. Our findings correspond to the hypothesis that muscular defects leading to impaired bowel integrity being an underlying etiology of SIP. In addition, histopathologic analysis revealed absence or thinning of the muscularis propria in 30% of SIP patients which further supports our molecular findings [29,30].

The present knowledge on the expressional pattern and the role of miRNAs in both non-inflammatory neonatal and diseased bowel tissues is very limited. Thus far, miRNA microarray studies have been reported in adult IBD tissues, but candidate miRNAs identified by different reports were diversified and could be due to tissue variation or employment of different analytical platforms for measurement [10,13,16,18]. Although many IBD-associated miRNAs did not appear to be dysregulated in preterm NEC tissues, several miRNAs, including miR-223, miR-132, miR-146b-3p, miR-215, miR-375, miR-31 and miR-141, were regulated in both IBD and NEC. More importantly, other dysregulated miRNAs observed in our study, such as miR-451, miR-1290, miR-4725-3p, miR-4793-3p, miR-431 and miR-203, have not been previously reported in other inflammatory intestinal conditions. Further, the specific role of an individual miRNA might be different between adult and neonatal inflammatory diseases. miR-31, a reported target of *hif1a*, was increased in specimens obtained from patients with active ulcerative colitis [31] and during progression and neoplastic transformation of IBD, [32] but it was down-regulated in NEC tissues (0.12 fold). Our network analysis also revealed possible association of miR-146-3p with arginine metabolism (*CPS1*) and G-protein mediated muscle contraction (*GNA11*), (Fig 3). Huang *et al*. proposed that miR-141 targeting *CXCL12B* might contribute to the development of intestinal inflammation by recruiting inflammatory cells in mice having colitis [26]. In NEC tissues, we showed that down-regulation of miR-141 could activate ECM remodeling (*TIMP1*, *SERPINE1*) and angiogenesis (*FLT1*, *THBS1*). Also, miR-132 was up-regulated in inflamed IBD tissues and suggested to play an inflammation-dependent homeostatic role on cholinergic signaling [15]. In our study, miR-132 exhibited inverse correlation with putative targets *HBEGF*, *CD44* and *MMP9* in NEC tissues. Overall, our observation indicated important roles of specific miRNAs in inflammation and maintenance of gut integrity, but their underlying involvement in the pathophysiology of neonatal NEC and adult IBD could be different, plausibly attributed to distinct target mRNAs in different disease conditions.

There are limitations in the current study. In the stringent selection of miRNA and potential target mRNA pairs, we have excluded those which demonstrated same directional changes, and/or those with significant positive correlation within same tissues (*e*.*g*., miR-410/*FLT1*; Fig 2 and S4 Table), as their involvement in the expression cascade would be difficult to interpret. It is possible that these miRNAs might have regulated their respective mRNAs indirectly through multiple intermediates of the expression cascade and novel mechanisms such as cell cycle-dependent regulatory switch [33–35]. Disputably, our network analysis was not extended to cover the association of target miRNAs outside the scope of the 52 validated mRNAs. Nonetheless, these mRNAs have been recognized to play key roles in the regulatory networks of NEC and SIP, and have definitive pathogenic and pathophysiologic relevance to these diseases [4]. Another intrinsic limitation of the study design was the difficulty in recruiting comparable gestational and postnatal age-matched control subjects. It is because no other common conditions in preterm infants would require bowel surgery during this postnatal period. To address this issue, we purposefully included Surg-CTL infants who underwent surgery for non-inflammatory conditions. Our rationale for targeting these control subjects was also supported by our previous observations that intense inflammatory responses occurred in NEC compared with SIP or Surg-CTL tissues, whereas SIP and Surg-CTL tissues exhibited minimal inflammatory response despite their differences in gestational age [4]. In view of the numerous and diversified target genes of a specific miRNA and its tissue- and disease-specific mechanisms, we anticipate conducting further experiments to understand the involvement of these miRNAs in NEC and SIP pathophysiology by *in vitro* cellular studies and *in vivo* animal models.

In summary, our study provides the first informational platform for identifying miRNAs and their novel roles in the neonatal gut of NEC and SIP. Dysregulation of these miRNAs could be mediated *via* microbial stimulation or hypoxic/oxidative insults, which triggered a concerted response across cell barriers leading to extensive inflammation and disruption of mucosal integrity. As specific miRNAs have been targeted for therapeutic applications in different diseases, [6,7] chemically engineered anti-miRNAs, miRNA mimics and their delivery strategies are currently under intensive investigation in animal studies and human efficacy trials [36]. A recent study has shown that treatment with miR-141 was effective in reducing intestinal inflammation in the mouse model of colitis [26]. As miRNAs are detectable in the circulation, miRNA-based diagnostics have also been explored, and to date much work has been performed on detecting and monitoring organ injury [37]. Our results have revealed coordinated dysregulatory networks and potential roles of the miRNA inhibitory mechanism in NEC and SIP. If proven effective at multiple hierarchies of the inflammatory cascade, specific miRNAs such as miR-31, miR-203 or miR-194-3p, could be developed for treatment to dampen the exaggerated immunologic response that contributes to the pathophysiology of NEC. Alternatively, specific miRNAs such as miR-146b-3p and miR-451 could be utilized to enhance intestinal muscular function in NEC and SIP. Importantly, miRNAs released from gut tissues to the circulation or gut lumen could also be used as disease-specific or organ-specific biomarkers for early diagnosis and prediction of outcomes for NEC, SIP and intestinal injury.

## Supporting Information

S1 Fig Selection of Reference Gene for qPCR Analysis of miRNAs. (PDF)

S1 Table A List of qPCR Reference Sequence Number and ID. (PDF)

S2 Table Microarray Data of miRNAs in Small Bowel Tissues from Infants with NEC, SIP and Surg-CTL. (PDF)

S3 Table A List of Dysregulated miRNAs and Proposed mRNA Targets and Functions. (PDF)

S4 Table Target mRNA Prediction of Differentially Expressed miRNAs in NEC or SIP Tissues by qPCR. (PDF)

## References

[1] Lin PW, Stoll BJ. Necrotising enterocolitis. Lancet. 2006; 368: 1271–1283. 17027734 10.1016/S0140-6736(06)69525-1

[2] Neu J, Walker WA. Necrotizing enterocolitis. N Engl J Med. 2011; 364: 255–264. 10.1056/NEJMra1005408 21247316 PMC3628622

[3] Pumberger W, Mayr M, Kohlhauser C, Weninger M. Spontaneous localized intestinal perforation in very-low-birth-weight infants: a distinct clinical entity different from necrotizing enterocolitis. J Am Coll Surg. 2002; 195: 796–803. 12495312 10.1016/s1072-7515(02)01344-3

[4] Chan KY, Leung KT, Tam YH, Lam HS, Cheung HM, Ma TP, et al Genome-wide expression profiles of necrotizing enterocolitis versus spontaneous intestinal perforation in human intestinal tissues: dysregulation of functional pathways. Ann Surg. 2014; 260: 1128–1137. 24368664 10.1097/SLA.0000000000000374

[5] Fabbri M, Croce CM, Calin GA. MicroRNAs. Cancer J. 2008; 14: 1–6. 18303474 10.1097/PPO.0b013e318164145e

[6] van RE, Purcell AL, Levin AA. Developing microRNA therapeutics. Circ Res. 2012; 110: 496–507. 10.1161/CIRCRESAHA.111.247916 22302756

[7] Broderick JA, Zamore PD. MicroRNA therapeutics. Gene Ther. 2011; 18: 1104–1110. 10.1038/gt.2011.50 21525952 PMC3237828

[8] Kozomara A, Griffiths-Jones S. miRBase: annotating high confidence microRNAs using deep sequencing data. Nucleic Acids Res. 2014; 42: D68–D73. 10.1093/nar/gkt1181 24275495 PMC3965103

[9] Rossi S, Tsirigos A, Amoroso A, Mascellani N, Rigoutsos I, Calin GA, et al OMiR: Identification of associations between OMIM diseases and microRNAs. Genomics. 2011; 97: 71–76. 10.1016/j.ygeno.2010.10.004 20974243

[10] Pekow JR, Kwon JH. MicroRNAs in inflammatory bowel disease. Inflamm Bowel Dis. 2012; 18: 187–193. 21425211 10.1002/ibd.21691 PMC4169145

[11] Bhalala OG, Srikanth M, Kessler JA. The emerging roles of microRNAs in CNS injuries. Nat Rev Neurol. 2013; 9: 328–339. 10.1038/nrneurol.2013.67 23588363 PMC3755895

[12] Shrivastava S, Petrone J, Steele R, Lauer GM, Di Bisceglie AM, Ray RB. Up-regulation of circulating miR-20a is correlated with hepatitis C virus-mediated liver disease progression. Hepatology. 2013; 58: 863–871. 10.1002/hep.26296 23390075 PMC3664107

[13] Coskun M, Bjerrum JT, Seidelin JB, Nielsen OH. MicroRNAs in inflammatory bowel disease—pathogenesis, diagnostics and therapeutics. World J Gastroenterol. 2012; 18: 4629–4634. 23002331 10.3748/wjg.v18.i34.4629 PMC3442200

[14] Koukos G, Polytarchou C, Kaplan JL, Morley-Fletcher A, Gras-Miralles B, Kokkotou E, et al MicroRNA-124 regulates STAT3 expression and is down-regulated in colon tissues of pediatric patients with ulcerative colitis. Gastroenterology. 2013; 145: 842–852. 23856509 10.1053/j.gastro.2013.07.001 PMC4427058

[15] Maharshak N, Shenhar-Tsarfaty S, Aroyo N, Orpaz N, Guberman I, Canaani J, et al MicroRNA-132 modulates cholinergic signaling and inflammation in human inflammatory bowel disease. Inflamm Bowel Dis. 2013; 19: 1346–1353. 23598815 10.1097/MIB.0b013e318281f47d

[16] Paraskevi A, Theodoropoulos G, Papaconstantinou I, Mantzaris G, Nikiteas N, Gazouli M. Circulating MicroRNA in inflammatory bowel disease. J Crohns Colitis. 2012; 6: 900–904. 10.1016/j.crohns.2012.02.006 22386737

[17] Wu F, Zikusoka M, Trindade A, Dassopoulos T, Harris ML, Bayless TM, et al MicroRNAs are differentially expressed in ulcerative colitis and alter expression of macrophage inflammatory peptide-2 alpha. Gastroenterology. 2008; 135: 1624–1635. 18835392 10.1053/j.gastro.2008.07.068

[18] Iborra M, Bernuzzi F, Correale C, Vetrano S, Fiorino G, Beltran B, et al Identification of serum and tissue micro-RNA expression profiles in different stages of inflammatory bowel disease. Clin Exp Immunol. 2013; 173: 250–258. 10.1111/cei.12104 23607522 PMC3722925

[19] Zahm AM, Hand NJ, Tsoucas DM, Le Guen CL, Baldassano RN, Friedman JR. Rectal microRNAs are perturbed in pediatric inflammatory bowel disease of the colon. J Crohns Colitis. 2014; 8: 1108–1117. 10.1016/j.crohns.2014.02.012 24613022 PMC4146627

[20] Schwarzenbach H, Nishida N, Calin GA, Pantel K. Clinical relevance of circulating cell-free microRNAs in cancer. Nat Rev Clin Oncol. 2014; 11: 145–156. 10.1038/nrclinonc.2014.5 24492836

[21] Paraskevopoulou MD, Georgakilas G, Kostoulas N, Vlachos IS, Vergoulis T, Reczko M, et al DIANA-microT web server v5.0: service integration into miRNA functional analysis workflows. Nucleic Acids Res. 2013; 41: W169–W173. 10.1093/nar/gkt393 23680784 PMC3692048

[22] Vergoulis T, Vlachos IS, Alexiou P, Georgakilas G, Maragkakis M, Reczko M, et al TarBase 6.0: capturing the exponential growth of miRNA targets with experimental support. Nucleic Acids Res. 2012; 40: D222–D229. 10.1093/nar/gkr1161 22135297 PMC3245116

[23] Lewis BP, Burge CB, Bartel DP. Conserved seed pairing, often flanked by adenosines, indicates that thousands of human genes are microRNA targets. Cell. 2005; 120: 15–20. 15652477 10.1016/j.cell.2004.12.035

[24] Wei J, Huang X, Zhang Z, Jia W, Zhao Z, Zhang Y, et al MyD88 as a target of microRNA-203 in regulation of lipopolysaccharide or Bacille Calmette-Guerin induced inflammatory response of macrophage RAW264.7 cells. Mol Immunol. 2013; 55: 303–309. 10.1016/j.molimm.2013.03.004 23522925

[25] Murata K, Yoshitomi H, Furu M, Ishikawa M, Shibuya H, Ito H, et al MicroRNA-451 down-regulates neutrophil chemotaxis via p38 MAPK. Arthritis Rheumatol. 2014; 66: 549–559. 10.1002/art.38269 24574214

[26] Huang Z, Shi T, Zhou Q, Shi S, Zhao R, Shi H, et al miR-141 Regulates colonic leukocytic trafficking by targeting CXCL12beta during murine colitis and human Crohn's disease. Gut. 2014; 63: 1247–1257. 10.1136/gutjnl-2012-304213 24000293

[27] Rangrez AY, M'Baya-Moutoula E, Metzinger-Le M V, Henaut L, Djelouat MS, Benchitrit J, et al Inorganic phosphate accelerates the migration of vascular smooth muscle cells: evidence for the involvement of miR-223. PLoS One. 2012; 7: e47807 10.1371/journal.pone.0047807 23094093 PMC3475714

[28] Dmitriev P, Barat A, Polesskaya A, O'Connell MJ, Robert T, Dessen P, et al Simultaneous miRNA and mRNA transcriptome profiling of human myoblasts reveals a novel set of myogenic differentiation-associated miRNAs and their target genes. BMC Genomics. 2013; 14: 265 10.1186/1471-2164-14-265 23597168 PMC3639941

[29] Holland AJ, Shun A, Martin HC, Cooke-Yarborough C, Holland J. Small bowel perforation in the premature neonate: congenital or acquired? Pediatr Surg Int. 2003; 19: 489–494. 12748799 10.1007/s00383-003-0967-8

[30] Kubota A, Yamanaka H, Okuyama H, Shiraishi J, Kawahara H, Hasegawa T, et al Focal intestinal perforation in extremely-low-birth-weight neonates: etiological consideration from histological findings. Pediatr Surg Int. 2007; 23: 997–1000. 17653555 10.1007/s00383-007-1984-9

[31] Fasseu M, Treton X, Guichard C, Pedruzzi E, Cazals-Hatem D, Richard C, et al Identification of restricted subsets of mature microRNA abnormally expressed in inactive colonic mucosa of patients with inflammatory bowel disease. PLoS One. 2010; 5: e13160 10.1371/journal.pone.0013160 20957151 PMC2950152

[32] Olaru AV, Cheng Y, Agarwal R, Yang J, David S, Abraham JM, et al Unique patterns of CpG island methylation in inflammatory bowel disease-associated colorectal cancers. Inflamm Bowel Dis. 2012; 18: 641–648. 21830278 10.1002/ibd.21826 PMC3214229

[33] Pasquinelli AE. MicroRNAs and their targets: recognition, regulation and an emerging reciprocal relationship. Nat Rev Genet. 2012; 13: 271–282. 10.1038/nrg3162 22411466

[34] Inui M, Martello G, Piccolo S. MicroRNA control of signal transduction. Nat Rev Mol Cell Biol. 2010; 11: 252–263. 10.1038/nrm2868 20216554

[35] Vasudevan S, Tong Y, Steitz JA. Switching from repression to activation: microRNAs can up-regulate translation. Science. 2007; 318: 1931–1934. 18048652 10.1126/science.1149460

[36] Chen N, Wang J, Hu Y, Cui B, Li W, Xu G, et al MicroRNA-410 reduces the expression of vascular endothelial growth factor and inhibits oxygen-induced retinal neovascularization. PLoS One. 2014; 9: e95665 10.1371/journal.pone.0095665 24777200 PMC4002426

[37] Laterza OF, Lim L, Garrett-Engele PW, Vlasakova K, Muniappa N, Tanaka WK, et al Plasma MicroRNAs as sensitive and specific biomarkers of tissue injury. Clin Chem. 2009; 55: 1977–1983. 10.1373/clinchem.2009.131797 19745058

