# Supplementary material for: Comparative MiRNA Expressional Profiles and Molecular Networks in Human Small Bowel Tissues of Necrotizing Enterocolitis and Spontaneous Intestinal Perforation
Source: PLoS One. 2015 Aug 14;10(8):e0135737. doi: 10.1371/journal.pone.0135737 (PMC4537110; doi:10.1371/journal.pone.0135737)
Supplement: S1 Fig — (PDF) [file pone.0135737.s001.pdf]

S1 Figure. Selection of Reference Gene for qPCR Analysis of miRNAs

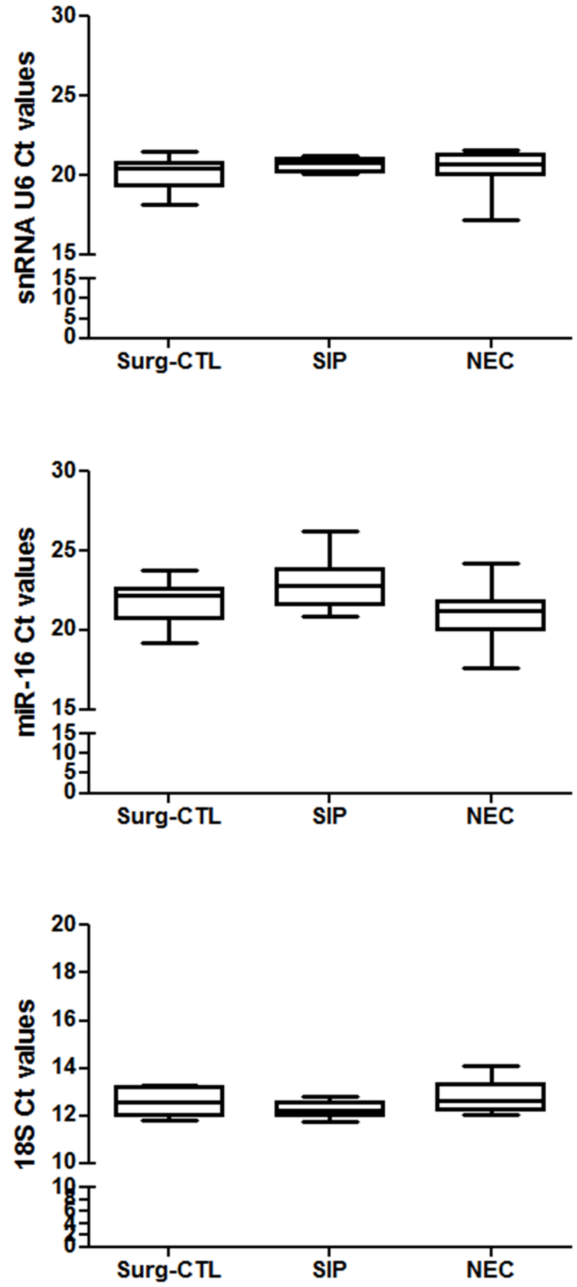

S1 Figure. Selection of Reference Gene for qPCR Analysis of miRNAs. Three reference genes snRNA U6, miR-16 and 18S RNA were compared in Surg-CTL, SIP and NEC samples (n=10 for each group). U6 was selected as the reference gene because of its consistency among groups (*P*=0.38, Kruskal Wallis test) and similarity in the cycle number and preparation method with target miRNAs. Results are expressed as median and interquartile range.
