# Supplementary material for: Comparative MiRNA Expressional Profiles and Molecular Networks in Human Small Bowel Tissues of Necrotizing Enterocolitis and Spontaneous Intestinal Perforation
Source: PLoS One. 2015 Aug 14;10(8):e0135737. doi: 10.1371/journal.pone.0135737 (PMC4537110; doi:10.1371/journal.pone.0135737)
Supplement: S1 Table — (PDF) [file pone.0135737.s002.pdf]

S1 Table. A List of qPCR Reference Sequence Number and ID

Gene Name	Assay ID	Accession No.
hsa-miR-1	002222	MIMAT0000416
hsa-miR-1231	002762	MIMAT0005586
hsa-miR-1290	002863	MIMAT0005880
hsa-miR-132	000457	MIMAT0000426
hsa-miR-133b	002247	MIMAT0000770
hsa-miR-141	000463	MIMAT0000432
hsa-miR-146b-3p	002361	MIMAT0004766
hsa-miR-187	001193	MIMAT0000262
hsa-miR-192-3p	002272	MIMAT0004543
hsa-miR-194-3p	002379	MIMAT0004671
hsa-miR-200a	000502	MIMAT0000682
hsa-miR-200b-5p	002274	MIMAT0004571
hsa-miR-203	000507	MIMAT0000264
hsa-miR-21-3p	002438	MIMAT0004494
hsa-miR-215	000518	MIMAT0000272
hsa-miR-223	002295	MIMAT0000280
hsa-miR-23b-5p	002126	MIMAT0004587
hsa-miR-31	002279	MIMAT0000089
hsa-miR-375	000564	MIMAT0000728
hsa-miR-410	001274	MIMAT0002171
hsa-miR-429	001024	MIMAT0001536
hsa-miR-4440	464070_mat	MIMAT0018958
hsa-miR-451	001141	MIMAT0001631
hsa-miR-4725-3p	463784_mat	MIMAT0019844
hsa-miR-4793-3p	463822_mat	MIMAT0019966
hsa-miR-490	001037	MIMAT0002806
hsa-miR-602	002925	MIMAT0003270
U6 snRNA	001973	NR_004394
