# Supplementary material for: Comparative MiRNA Expressional Profiles and Molecular Networks in Human Small Bowel Tissues of Necrotizing Enterocolitis and Spontaneous Intestinal Perforation
Source: PLoS One. 2015 Aug 14;10(8):e0135737. doi: 10.1371/journal.pone.0135737 (PMC4537110; doi:10.1371/journal.pone.0135737)
Supplement: S2 Table — (PDF) [file pone.0135737.s003.pdf]

S2 Table. Microarray Data of miRNAs in Small Bowel Tissues from Infants with NEC, SIP and Surg-CTL

	NEC vs Surg-CTL		SIP vs Surg-CTL		NEC vs SIP	
**miRNAs**	**Fold**	***P***	**Fold**	***P***	**Fold**	***P***
hsa-miR-223	**11.26**	**0.000**	**1.60**	0.177	**7.05**	**0.000**
hsa-miR-451	2.99	**0.013**	2.14	0.063	1.40	0.376
hsa-miR-1290	33.22	**0.021**	0.69	0.773	48.25	**0.013**
hsa-miR-4725-3p	4.06	0.075	7.57	**0.017**	0.54	0.393
hsa-miR-431	6.04	**0.015**	4.81	**0.027**	1.26	0.712
hsa-miR-4793-3p	12.23	**0.007**	3.03	0.160	4.04	0.085
hsa-miR-21-3p	6.10	**0.002**	2.05	0.115	2.98	**0.026**
hsa-miR-132	3.48	**0.000**	2.18	**0.008**	1.60	0.068
hsa-miR-146b-3p	5.34	**0.014**	2.48	0.132	2.16	0.194
hsa-miR-410	4.47	**0.003**	1.23	0.587	3.64	**0.006**
hsa-miR-429	0.33	**0.000**	3.20	**0.000**	0.10	**0.000**
hsa-miR-187	0.91	0.917	0.12	**0.047**	7.69	0.056
hsa-miR-375	0.01	**0.000**	1.28	0.726	0.01	**0.000**
hsa-miR-203	0.03	**0.000**	1.10	0.780	0.02	**0.000**
hsa-miR-200b-5p	0.01	**0.000**	0.98	0.972	0.01	**0.000**
hsa-miR-194-3p	0.01	**0.000**	1.01	0.975	0.01	**0.000**
hsa-miR-200a	0.01	**0.000**	1.42	0.483	0.01	**0.000**
hsa-miR-215	0.01	**0.000**	1.37	0.653	0.01	**0.000**
hsa-miR-31	0.05	**0.000**	0.97	0.946	0.05	**0.000**
hsa-miR-192-3p	0.01	**0.000**	1.37	0.324	0.01	**0.000**
hsa-miR-141	0.01	**0.000**	1.32	0.461	0.01	**0.000**
hsa-miR-1231	2.66	**0.039**	5.13	**0.003**	0.52	0.140
hsa-miR-1	0.69	0.258	0.25	**0.001**	3.04	**0.006**
hsa-miR-602	1.93	**0.022**	3.69	**0.000**	0.52	**0.023**
hsa-miR-4440	6.69	**0.006**	2.47	0.125	2.71	0.095
hsa-miR-133b	0.84	0.602	0.24	**0.001**	3.55	**0.003**
hsa-miR-23b-5p	0.61	0.260	0.17	**0.002**	3.55	**0.013**
hsa-miR-490-3p	0.65	0.360	0.22	**0.008**	2.93	**0.038**

Bold values indicate statistically significant comparisons. The microarray data sets were submitted to Gene Expression Omnibus database (GSE68054).
