# Supplementary material for: Comparative MiRNA Expressional Profiles and Molecular Networks in Human Small Bowel Tissues of Necrotizing Enterocolitis and Spontaneous Intestinal Perforation
Source: PLoS One. 2015 Aug 14;10(8):e0135737. doi: 10.1371/journal.pone.0135737 (PMC4537110; doi:10.1371/journal.pone.0135737)
Supplement: S3 Table — (PDF) [file pone.0135737.s004.pdf]

S3 Table. A List of Dysregulated miRNAs and Proposed mRNA Targets and Functions

miRNAs	miRBase ID	Clusters	Chromosome location	Direct and indirect target mRNAs	Functions	Ref
miR-223	hsa-miR-223-3p		chrX	IκB kinase subunit alpha (*CHUK*)	-monocyte/macrophage -differentiation, NF-κB pathway	[1,2]
				NFIA	-granulocytic differentiation	[3]
				NLRP3	-inflammation, IL1B production	[4]
				ICAM1	-anti-inflammatory response	[5]
				Data not available	-dysregulated in IBD	[6,7]
miR-451	hsa-miR-451a	451a/451b	chr17	*CPNE3*, *RAB5A*	-neutrophil chemotaxis	[8]
miR-1290	hsa-miR-1290			*FOXA1*	-cell cycle and differentiation, gastric epithelial cells	[9]
				NKRF, *NIF13B*	-NF-κB activation (colon cancer)	[10]
miR-4725-3p	hsa-miR-4725-3p		chr17	Data not available	Data not available	
miR-431	has-miR-431-5p	493/337/431/433/136	chr14	reduced *IGF1R* and *IRS2* expression	-inhibition of cell proliferation by suppressing the MAPK pathway	[11]
				reduced *SOCS6* expression	-cell viability (cancer)	[12]
miR-4793-3p	hsa-miR-4793-3p		chr3	Data not available	Data not available	
miR-21-3p	hsa-miR-21-3p		chr17	Data not available	Data not available	
miR-132	hsa-miR-132-3p	132/212	chr17	*ACHE*	-cholinergic signaling, anti-inflammation	[13,14]
				HBEGF	-negative feedback mechanism of mast cell activation	[15]
				IRAK4	-TLR2-mediated tolerance and cross-tolerance in THP-1 monocytes	[16]
miR-146b-3p	hsa-miR-146b-3p		chr10	Data not available	-dysregulated in IBD	[17]
miR-410	hsa-miR-410-3p	379/410	chr14	*VEGFA*	-anti-angiogenesis	[18]
miR-429	hsa-miR-429	200a/200b/429	chr1	AP-2apha (*TFAP2A*)	-cell proliferation and apoptosis (cancer)	[19]
miR-187	hsa-miR-187-3p		chr18	*HIPK3*	-regulation of insulin secretion	[20]
miR-375	hsa-miR-375		chr2	AEG-1 (*MTDH*)	-suppressing cell viability and clonogenic survival, cell	[21]

					migration/invasion, as well as in vivo tumor formation (cancer)	
				hnf6, *insm1*, *ngn3*, *neurod1*, and *pdx1*	-controlling cellular growth and proliferation in the developing pancreas (rodent)	[22]
				Data not available	-dysregulation in IBD	[23]
miR-203	hsa-miR-203a-3p	203a/203b	chr14	*SOCS3*	-STAT3 signaling	[24]
				SOCS6, *TNF* and *IL24*	-anti-inflammation	[25]
				IL8	- regulator of keratinocyte differentiation	[26]
				myd88	- toll-like receptors signaling (rodent)	[27]
miR-200b-5p	hsa-miR-200b-5p	200b/200a/ 429	chr1	Data not available	Data not available	
miR-194-3p	hsa-miR-194-3p	194-1/215	chr1	Data not available	Data not available	
		192/194-2	chr11			
miR-200a	hsa-miR-200a-3p	200b/200a/ 429	chr1	*ZEB1*, *ZEB2*/*SIP1*	-maintenance of the epithelial phenotype	[28,29]
				THBS1	-HUVEC viability, angiogenesis	[30]
				IL8	-regulation of angiogenesis	[31]
				PTEN	-cell migration and proliferation	[32]
				reduced *ZEB1*expression	-colon cancer cells (cancer)	[33,34]
miR-215	hsa-miR-215-5p	194-1/215	chr1	*ZEB2*/*SIP1*	-induction of E-cadherin expression	[35]
				Data not available	-dysregulated in IBD	[7]
miR-31	hsa-miR-31-5p			*hif1a*	-ischemia reduces miR-31 in mice retina, regulates angiogenesis via VEGFA (rodent)	[36]
				RHOA	-IL-2 production during T cell activation, inflammation	[37]
				Data not available	-dysregulated in IBD	[7,38]
miR-192-3p	hsa-miR-192-3p	194-2/192	chr11	Data not available	Data not available	
miR-141	hsa-miR-141-3p	200c/141	chr12	*CD47*, *CUL3*, *PTEN*	-cell migration and proliferation	[32,39]
				CXCL12B	-leukocyte migration	[40]

Supplemental References

1. Li T, Morgan MJ, Choksi S, Zhang Y, Kim YS, Liu ZG (2010) MicroRNAs modulate the noncanonical transcription factor NF-kappaB pathway by regulating expression of the kinase IKKalpha during macrophage differentiation. Nat Immunol 11: 799-805. doi:ni.1918 [pii];10.1038/ni.1918 [doi] PMC2926307

2. O'Connell RM, Zhao JL, Rao DS (2011) MicroRNA function in myeloid biology. Blood 118: 2960-2969. doi:blood-2011-03-291971 [pii];10.1182/blood-2011-03-291971 [doi] PMC3175776

3. Fazi F, Rosa A, Fatica A, Gelmetti V, De Marchis ML, Nervi C, et al. (2005) A minicircuitry comprised of microRNA-223 and transcription factors NFI-A and C/EBPalpha regulates human granulopoiesis. Cell 123: 819-831. doi:S0092-8674(05)00977-3 [pii];10.1016/j.cell.2005.09.023 [doi]

4. Bauernfeind F, Rieger A, Schildberg FA, Knolle PA, Schmid-Burgk JL, Hornung V (2012) NLRP3 inflammasome activity is negatively controlled by miR-223. J Immunol 189: 4175-4181. doi:jimmunol.1201516 [pii];10.4049/jimmunol.1201516 [doi]

5. Tabet F, Vickers KC, Cuesta Torres LF, Wiese CB, Shoucri BM, Lambert G, et al. (2014) HDL-transferred microRNA-223 regulates ICAM-1 expression in endothelial cells. Nat Commun 5: 3292. doi:ncomms4292 [pii];10.1038/ncomms4292 [doi] PMC4189962

6. Wu F, Zhang S, Dassopoulos T, Harris ML, Bayless TM, Meltzer SJ, et al. (2010) Identification of microRNAs associated with ileal and colonic Crohn's disease. Inflamm Bowel Dis 16: 1729-1738. doi:10.1002/ibd.21267 [doi] PMC2946509

7. Fasseu M, Treton X, Guichard C, Pedruzzi E, Cazals-Hatem D, Richard C, et al. (2010) Identification of restricted subsets of mature microRNA abnormally expressed in inactive colonic mucosa of patients with inflammatory bowel disease. PLoS One 5: e13160. doi:10.1371/journal.pone.0013160 [doi] PMC2950152

8. Murata K, Yoshitomi H, Furu M, Ishikawa M, Shibuya H, Ito H, et al. (2014) MicroRNA-451 down-regulates neutrophil chemotaxis via p38 MAPK. Arthritis Rheumatol 66: 549-559. doi:10.1002/art.38269 [doi]

9. Zhu Y, Jiang Q, Lou X, Ji X, Wen Z, Wu J, et al. (2012) MicroRNAs up-regulated by CagA of Helicobacter pylori induce intestinal metaplasia of gastric epithelial cells. PLoS One 7: e35147. doi:10.1371/journal.pone.0035147 [doi];PONE-D-12-00471 [pii] PMC3335061

10. Wu J, Ji X, Zhu L, Jiang Q, Wen Z, Xu S, et al. (2013) Up-regulation of microRNA-1290 impairs cytokinesis and affects the reprogramming of colon cancer cells. Cancer Lett 329: 155-163. doi:S0304-3835(12)00643-X [pii];10.1016/j.canlet.2012.10.038 [doi]

11. Tanaka T, Sugaya S, Kita K, Arai M, Kanda T, Fujii K, et al. (2012) Inhibition of cell viability by human IFN-beta is mediated by microRNA-431. Int J Oncol 40: 1470-1476. doi:10.3892/ijo.2012.1345 [doi]

12. Tanaka T, Arai M, Jiang X, Sugaya S, Kanda T, Fujii K, et al. (2014) Downregulation of microRNA-431 by human interferon-beta inhibits viability of medulloblastoma and glioblastoma cells via upregulation of SOCS6. Int J Oncol 44: 1685-1690. doi:10.3892/ijo.2014.2317 [doi]

13. Maharshak N, Shenhar-Tsarfaty S, Aroyo N, Orpaz N, Guberman I, Canaani J, et al. (2013) MicroRNA-132 modulates cholinergic signaling and inflammation in human inflammatory bowel disease. Inflamm Bowel Dis 19: 1346-1353. doi:10.1097/MIB.0b013e318281f47d [doi]

14. Shaked I, Meerson A, Wolf Y, Avni R, Greenberg D, Gilboa-Geffen A, et al. (2009) MicroRNA-132 potentiates cholinergic anti-inflammatory signaling by targeting acetylcholinesterase. Immunity 31: 965-973. doi:S1074-7613(09)00496-8 [pii];10.1016/j.immuni.2009.09.019 [doi]

15. Molnar V, Ersek B, Wiener Z, Tombol Z, Szabo PM, Igaz P, et al. (2012) MicroRNA-132 targets HB-EGF upon IgE-mediated activation in murine and human mast cells. Cell Mol Life Sci 69: 793-808. doi:10.1007/s00018-011-0786-3 [doi]

16. Nahid MA, Yao B, Dominguez-Gutierrez PR, Kesavalu L, Satoh M, Chan EK (2013) Regulation of TLR2-mediated tolerance and cross-tolerance through IRAK4 modulation by miR-132 and miR-212. J Immunol 190: 1250-1263. doi:jimmunol.1103060 [pii];10.4049/jimmunol.1103060 [doi] PMC3552145

17. Coskun M, Bjerrum JT, Seidelin JB, Nielsen OH (2012) MicroRNAs in inflammatory bowel disease--pathogenesis, diagnostics and therapeutics. World J Gastroenterol 18: 4629-4634. doi:10.3748/wjg.v18.i34.4629 [doi] PMC3442200

18. Chen N, Wang J, Hu Y, Cui B, Li W, Xu G, et al. (2014) MicroRNA-410 reduces the expression of vascular endothelial growth factor and inhibits oxygen-induced retinal neovascularization. PLoS One 9: e95665. doi:10.1371/journal.pone.0095665 [doi];PONE-D-13-32217 [pii] PMC4002426

19. Wu Y, Xiao Y, Ding X, Zhuo Y, Ren P, Zhou C, et al. (2011) A miR-200b/200c/429-binding site polymorphism in the 3' untranslated region of the AP-2alpha gene is associated with cisplatin resistance. PLoS One 6: e29043. doi:10.1371/journal.pone.0029043 [doi];PONE-D-11-14422 [pii] PMC3237583

20. Locke JM, da S, X, Dawe HR, Rutter GA, Harries LW (2014) Increased expression of miR-187 in human islets from individuals with type 2 diabetes is associated with reduced glucose-stimulated insulin secretion. Diabetologia 57: 122-128. doi:10.1007/s00125-013-3089-4 [doi] PMC3855472

21. Nohata N, Hanazawa T, Kikkawa N, Mutallip M, Sakurai D, Fujimura L, et al. (2011) Tumor suppressive microRNA-375 regulates oncogene AEG-1/MTDH in head and neck squamous cell carcinoma (HNSCC). J Hum Genet 56: 595-601. doi:jhg201166 [pii];10.1038/jhg.2011.66 [doi]

22. Li X (2014) MiR-375, a microRNA related to diabetes. Gene 533: 1-4. doi:S0378-1119(13)01339-5 [pii];10.1016/j.gene.2013.09.105 [doi]

23. Wu F, Zikusoka M, Trindade A, Dassopoulos T, Harris ML, Bayless TM, et al. (2008) MicroRNAs are differentially expressed in ulcerative colitis and alter expression of macrophage inflammatory peptide-2 alpha. Gastroenterology 135: 1624-1635. doi:S0016-5085(08)01408-X [pii];10.1053/j.gastro.2008.07.068 [doi]

24. Moffatt CE, Lamont RJ (2011) Porphyromonas gingivalis induction of microRNA-203 expression controls suppressor of cytokine signaling 3 in gingival epithelial cells. Infect Immun 79: 2632-2637. doi:IAI.00082-11 [pii];10.1128/IAI.00082-11 [doi] PMC3191996

25. Primo MN, Bak RO, Schibler B, Mikkelsen JG (2012) Regulation of pro-inflammatory cytokines TNFalpha and IL24 by microRNA-203 in primary keratinocytes. Cytokine 60: 741-748. doi:S1043-4666(12)00613-8 [pii];10.1016/j.cyto.2012.07.031 [doi]

26. Wei T, Xu N, Meisgen F, Stahle M, Sonkoly E, Pivarcsi A (2013) Interleukin-8 is regulated by miR-203 at the posttranscriptional level in primary human keratinocytes. Eur J Dermatol . doi:ejd.2013.1997 [pii];10.1684/ejd.2013.1997 [doi]

27. Wei J, Huang X, Zhang Z, Jia W, Zhao Z, Zhang Y, et al. (2013) MyD88 as a target of microRNA-203 in regulation of lipopolysaccharide or Bacille Calmette-Guerin induced inflammatory response of macrophage RAW264.7 cells. Mol Immunol 55: 303-309. doi:S0161-5890(13)00064-3 [pii];10.1016/j.molimm.2013.03.004 [doi]

28. Zhang B, Zhang Z, Xia S, Xing C, Ci X, Li X, et al. (2013) KLF5 activates microRNA 200 transcription to maintain epithelial characteristics and prevent induced epithelial-mesenchymal transition in epithelial cells. Mol Cell Biol 33: 4919-4935. doi:MCB.00787-13 [pii];10.1128/MCB.00787-13 [doi] PMC3889554

29. Gregory PA, Bert AG, Paterson EL, Barry SC, Tsykin A, Farshid G, et al. (2008) The miR-200 family and miR-205 regulate epithelial to mesenchymal transition by targeting ZEB1 and SIP1. Nat Cell Biol 10: 593-601. doi:ncb1722 [pii];10.1038/ncb1722 [doi]

30. Li YX, Liu DQ, Zheng C, Zheng SQ, Liu M, Li X, et al. (2011) miR-200a modulate HUVECs viability and migration. IUBMB Life 63: 553-559. doi:10.1002/iub.486 [doi]

31. Pecot CV, Rupaimoole R, Yang D, Akbani R, Ivan C, Lu C, et al. (2013) Tumour angiogenesis regulation by the miR-200 family. Nat Commun 4: 2427. doi:ncomms3427 [pii];10.1038/ncomms3427 [doi] PMC3904438

32. Li H, Tang J, Lei H, Cai P, Zhu H, Li B, et al. (2014) Decreased MiR-200a/141 suppress cell migration and proliferation by targeting PTEN in Hirschsprung's disease. Cell Physiol Biochem 34: 543-553. doi:000363021 [pii];10.1159/000363021 [doi]

33. Edvardsson K, Nguyen-Vu T, Kalasekar SM, Ponten F, Gustafsson JA, Williams C (2013) Estrogen receptor beta expression induces changes in the microRNA pool in human colon cancer cells. Carcinogenesis 34: 1431-1441. doi:bgt067 [pii];10.1093/carcin/bgt067 [doi]

34. Chen Y, Ge W, Xu L, Qu C, Zhu M, Zhang W, et al. (2012) miR-200b is involved in intestinal fibrosis of Crohn's disease. Int J Mol Med 29: 601-606. doi:10.3892/ijmm.2012.894 [doi] PMC3573760

35. Wang B, Herman-Edelstein M, Koh P, Burns W, Jandeleit-Dahm K, Watson A, et al. (2010) E-cadherin expression is regulated by miR-192/215 by a mechanism that is independent of the profibrotic effects of transforming growth factor-beta. Diabetes 59: 1794-1802. doi:db09-1736 [pii];10.2337/db09-1736 [doi] PMC2889781

36. Shen J, Yang X, Xie B, Chen Y, Swaim M, Hackett SF, et al. (2008) MicroRNAs regulate ocular neovascularization. Mol Ther 16: 1208-1216. doi:mt2008104 [pii];10.1038/mt.2008.104 [doi] PMC3033219

37. Fan W, Liang D, Tang Y, Qu B, Cui H, Luo X, et al. (2012) Identification of microRNA-31 as a novel regulator contributing to impaired interleukin-2 production in T cells from patients with systemic lupus erythematosus. Arthritis Rheum 64: 3715-3725. doi:10.1002/art.34596 [doi]

38. Paraskevi A, Theodoropoulos G, Papaconstantinou I, Mantzaris G, Nikiteas N, Gazouli M (2012) Circulating MicroRNA in inflammatory bowel disease. J Crohns Colitis 6: 900-904. doi:S1873-9946(12)00062-1 [pii];10.1016/j.crohns.2012.02.006 [doi]

39. Tang W, Qin J, Tang J, Zhang H, Zhou Z, Li B, et al. (2013) Aberrant reduction of MiR-141 increased CD47/CUL3 in Hirschsprung's disease. Cell Physiol Biochem 32: 1655-1667. doi:000356601 [pii];10.1159/000356601 [doi]

40. Huang Z, Shi T, Zhou Q, Shi S, Zhao R, Shi H, et al. (2014) miR-141 Regulates colonic leukocytic trafficking by targeting CXCL12beta during murine colitis and human Crohn's disease. Gut 63: 1247-1257. doi:gutjnl-2012-304213 [pii];10.1136/gutjnl-2012-304213 [doi]
