# Supplementary material for: Comparative MiRNA Expressional Profiles and Molecular Networks in Human Small Bowel Tissues of Necrotizing Enterocolitis and Spontaneous Intestinal Perforation
Source: PLoS One. 2015 Aug 14;10(8):e0135737. doi: 10.1371/journal.pone.0135737 (PMC4537110; doi:10.1371/journal.pone.0135737)
Supplement: S4 Table — (PDF) [file pone.0135737.s005.pdf]

S4 Table. Target mRNA Prediction of Differentially Expressed miRNAs in NEC or SIP Tissues by qPCR

**NEC**		
**miRNA (NEC vs Surg-CTL)**	**mRNA (NEC vs Surg-CTL)**	**Database**
miR-223 (↑)	*ICAM1* (↑)	DIANA-MicroT/ MetaCore
	MMP9 (↑)	MicroCosm
	IL6 (↑)	MetaCore
	NLRP3 (↑)	DIANA-MicroT/ MetaCore/MicroCosm
miR-451 (↑)	*OAT* (↓)	MicroCosm
	TLR4 (↑)	MicroCosm
	GNA11 (↓)	MicroCosm
miR-1290 (↑)	*THBS1* (↑)	DIANA-MicroT
	SOD2 (↑)	DIANA-MicroT
	NLRP3 (↑)	DIANA-MicroT
	CASQ2 (↓)	DIANA-MicroT
	FOXA1 (↓)	DIANA-MicroT/ MetaCore
miR-4725-3p (↑)	*TREM1* (↑)	DIANA-MicroT
	HMOX1 (↑)	DIANA-MicroT
	NR1H4 (↓)	DIANA-MicroT
	THBS1 (↑)	DIANA-MicroT
miR-431 (↑)	*FOXA1* (↓)	DIANA-MicroT
miR-4793-3p (↑)	*TLR4* (↑)	DIANA-MicroT/TargetScan
miR-21-3p (↑)	*MMP9* (↑)	MetaCore
	FOXA1 (↓)	DIANA-MicroT
miR-132 (↑)	*HBEGF* (↑)	DIANA-MicroT/ TargetScan/ MicroCosm/ Tarbase/ MetaCore
	THBS1 (↑)	Tarbase
	CD44 (↑)	DIANA-MicroT
	MMP9 (↑)	MetaCore
	PTGS2 (↑)	DIANA-MicroT/ Tarbase
	SOD2 (↑)	DIANA-MicroT
	IL6 (↑)	MicroCosm
miR-146b-3p (↑)	*CPS1* (↓)	DIANA-MicroT
	CD44 (↑)	MicroCosm
	IL6 (↑)	MicroCosm
	MEFV (↑)	DIANA-MicroT
	GNA11 (↓)	MicroCosm
	MYLK (↓)	DIANA-MicroT

miR-410 (↑)	*FLT1* (↑)	TargetScan
	HBEGF (↑)	TargetScan
	CXCL5 (↑)	DIANA-MicroT
	SERPINB2 (↑)	DIANA-MicroT/ TargetScan/MicroCosm
	HMOX1 (↑)	MicroCosm
	PTGS2 (↑)	DIANA-MicroT
miR-375 (↓)	*THBS1* (↑)	DIANA-MicroT
	FOXA1 (↓)	DIANA-MicroT
miR-203 (↓)	*HBEGF* (↑)	TargetScan
	PTGS2 (↑)	DIANA-MicroT
	IL8 (↑)	MetaCore
	NFKB2 (↑)	MetaCore
	TLR4 (↑)	DIANA-MicroT
	TNF (↑)	MetaCore
miR-200b-5p (↓)	*FLT1* (↑)	MicroCosm
	SOD2 (↑)	MicroCosm
miR-194-3p (↓)	*MMP9* (↑)	MicroCosm
	TIMP1 (↑)	MicroCosm
	FOSL1 (↑)	DIANA-MicroT/ MicroCosm
miR-200a (↓)	*ACE2* (↓)	MicroCosm
	THBS1 (↑)	MetaCore
	CPS1 (↓)	MicroCosm
	ICAM1 (↑)	MicroCosm
	TIMP1 (↑)	MicroCosm
	IL8 (↑)	MetaCore
miR-215 (↓)	*ANGPTL4* (↑)	MicroCosm
	NOD2 (↑)	MicroCosm
	MYLK (↓)	MetaCore
miR-31 (↓)	*HBEGF* (↑)	DIANA-MicroT/TargetScan
	HIF1A (↑)	MetaCore
	TLR4 (↑)	DIANA-MicroT
miR-192-3p (↓)	*NR1H4* (↓)	DIANA-MicroT/ MicroCosm
miR-141 (↓)	*ACE2* (↓)	MicroCosm
	FLT1 (↑)	DIANA-MicroT
	THBS1 (↑)	MetaCore
	CPS1 (↓)	DIANA-MicroT/ MicroCosm
	SERPINE1 (↑)	DIANA-MicroT
	TIMP1 (↑)	MicroCosm
	FOXA1 (↓)	DIANA-MicroT

SIP		
**miRNA (SIP vs Surg-CTL)**	**mRNA (SIP vs Surg-CTL)**	**Database**
miR-223 (↑)	*KCNMA1* (↓)	DIANA-MicroT
miR451 (↑)	*GNA11* (↓)	MicroCosm
	MYMO1 (↓)	MicroCosm
miR-429 (↑)	*TREM1* (↑)	DIANA-MicroT

The predicted lists were generated from 5 miRNA target prediction programs and publication database, DIANA-microT web server v5.0 (http://diana.imis.athena-innovation.gr/DianaTools/index.php?r=microT_CDS/index)[1], Tarbase 6.0 (http://diana.imis.athena-innovation.gr/DianaTools/index.php?r=tarbase/index) [2], TargetScan6.2 (www.targetscan.com)[3], MicroCosm (http://www.ebi.ac.uk/enright-srv/microcosm/htdocs/targets/v5/) and MetaCore.

Supplemental References

1. Paraskevopoulou MD, Georgakilas G, Kostoulas N, Vlachos IS, Vergoulis T, Reczko M, et al. (2013) DIANA-microT web server v5.0: service integration into miRNA functional analysis workflows. Nucleic Acids Res 41: W169-W173. doi:gkt393 [pii];10.1093/nar/gkt393 [doi] PMC3692048

2. Vergoulis T, Vlachos IS, Alexiou P, Georgakilas G, Maragkakis M, Reczko M, et al. (2012) TarBase 6.0: capturing the exponential growth of miRNA targets with experimental support. Nucleic Acids Res 40: D222-D229. doi:gkr1161 [pii];10.1093/nar/gkr1161 [doi] PMC3245116

3. Lewis BP, Burge CB, Bartel DP (2005) Conserved seed pairing, often flanked by adenosines, indicates that thousands of human genes are microRNA targets. Cell 120: 15-20. doi:S0092867404012607 [pii];10.1016/j.cell.2004.12.035 [doi]
